# Fasting vs. post-breakfast tabata exercise: implications for substrate metabolism and energy expenditure in young normal-weight women

**DOI:** 10.3389/fphys.2025.1721312

**Published:** 2026-01-16

**Authors:** Yongbo Wang, Yanbai Han, Zhuoyue Cheng, Yaqing Fan, Hongli Wang

**Affiliations:** College of Physical Education and Health, Guangxi Normal University, Guilin, China

**Keywords:** tabata, fasting, postprandial, fat oxidation, energy expenditure

## Abstract

**Introduction:**

With the rising prevalence of obesity, time-efficient high-intensity exercises like Tabata training have gained significant attention for weight management. However, the effects of fasting *versus* post-breakfast states on substrate metabolism and energy expenditure during Tabata exercise remain unclear. This study aimed to investigate the metabolic responses to Tabata exercise under fasting and post-breakfast conditions in women, providing insight into how nutritional status acutely influences substrate utilization and energy expenditure.

**Methods:**

Eighteen young normal-weight women (age 25.3 ± 3.1 years; BMI 20.9 ± 1.1 kg/m^2^)completed a randomized counterbalanced crossover trial, performing a 4-min Tabata workout under fasting (11–15 h overnight fast) and post-breakfast (90 min after a standardized meal) conditions. Gas exchange was continuously monitored to calculate fat oxidation, glucose oxidation, and energy expenditure.

**Results:**

Fat oxidation was significantly higher in the fasting condition at all analyzed time points, with the largest difference observed at 60 s (1.05 ± 0.18 vs. 0.61 ± 0.07 g/min, p < 0.001). In contrast, glucose oxidation was consistently higher in the post-breakfast condition, peaking at 150 s (3.65 ± 0.52 vs. 3.38 ± 0.46 g/min, p < 0.001). Total energy expenditure was also greater post-breakfast, reaching 10.18 ± 0.29 kcal/min at 120 s compared with 9.70 ± 0.39 kcal/min in the fasting condition (p < 0.001).

**Conclusion:**

Fasting and post-breakfast conditions elicit distinct acute metabolic responses during Tabata exercise in women. Fat oxidation was higher in the fasting state, while glucose oxidation and total energy expenditure were consistently higher in the post-breakfast state.

## 1 Introduction

In recent years, obesity has become a major public health concern worldwide, posing serious threats due to its strong association with metabolic disorders and cardiovascular diseases. With improvements in living standards and lifestyle changes, the prevalence of obesity continues to rise, thereby increasing the risk of chronic diseases and placing a heavy burden on healthcare systems. Developing scientifically effective strategies to enhance weight management and metabolic health has therefore become a central focus in the field of exercise science (NCD Risk Factor Collaboration, 2024; Hariharan et al., 2022).

Exercise is widely recognized as a cornerstone intervention for obesity management, with aerobic and resistance training being among the most commonly recommended modalities (Berge et al., 2021; Oppert et al., 2023). In recent years, Tabata training has gained considerable attention due to its time efficiency and minimal space requirements. Originating in Japan, the Tabata protocol consists of 20 s of high-intensity exercise followed by 10 s of rest, repeated for a total duration of 4 min. Compared with traditional moderate-intensity continuous training (MICT), Tabata training elicits greater energy expenditure per unit time and produces a pronounced acute excess post-exercise oxygen consumption (EPOC) response, reflecting sustained elevations in post-exercise metabolic rate (Børsheim and Bahr, 2023; LaForgia et al., 2006). These characteristics make Tabata a practical and time-efficient high-intensity interval training modality, particularly for individuals seeking short-duration exercise options (Lee et al., 2021; Tabata, 2019; Wang et al., 2024).

Although Tabata training has demonstrated significant metabolic benefits, its outcomes are strongly influenced by multiple factors, with nutritional status emerging as a critical determinant. In a fasting state, limited glucose availability prompts the body to rely more heavily on fat oxidation, which is considered beneficial for fat metabolism (Aird et al., 2018; Vieira et al., 2016; Wallis and Gonzalez, 2019). By contrast, in a postprandial state, elevated blood glucose and heightened insulin activity facilitate greater glucose oxidation, potentially supporting higher exercise intensity and overall energy expenditure. Importantly, not all postprandial states are metabolically equivalent: breakfast, consumed after an overnight fast, represents a unique physiological condition, characterized by relatively depleted glycogen stores, heightened insulin sensitivity, and a dynamic balance between fat and carbohydrate oxidation. Nutritional status, therefore, exerts a profound influence on substrate utilization through hormonal regulation involving insulin, catecholamines, and growth hormone (Papakonstantinou et al., 2022; Pearson et al., 2020).

Previous studies have confirmed that nutritional status significantly alters energy metabolism during moderate-intensity continuous training (MICT) and high-intensity interval training (HIIT) (Guo et al., 2023; Ryan et al., 2020). However, limited research has focused on the metabolic characteristics of Tabata training, a short-duration high-intensity protocol, under different feeding states. Post-breakfast Tabata exercise may differ from exercise following other meals. This study investigates fat oxidation, glucose oxidation, and energy expenditure during Tabata exercise in 18 adult women under fasting and post-breakfast conditions, aiming to enhance the understanding of acute metabolic responses to high-intensity interval exercise under different nutritional states.

## 2 Subjects and methods

This study employed a randomized counterbalanced crossover design involving 18 healthy young women. Each participant completed two Tabata exercise sessions under fasting (11–15 h overnight fast) and post-breakfast (90 min after a standardized meal) conditions. Participants were randomly assigned to one of two counterbalanced sequences (fasting→post-breakfast or post-breakfast→fasting) using a computer-generated randomization list, with a 7-day washout period between sessions. During each trial, real-time gas exchange parameters were continuously collected throughout the exercise to calculate fat oxidation, glucose oxidation, and energy expenditure.

### 2.1 Participants

Eighteen healthy adult women participated in the study (mean ± SD: age = 25.3 ± 3.1 years, height = 165.5 ± 5.8 cm, weight = 57.2 ± 4.6 kg, body fat percentage = 22.3 ± 2.1%, BMI = 20.9 ± 1.1 kg/m^2^). All participants were free from metabolic diseases and did not engage in regular structured exercise. Individuals who were regular smokers, who had diagnosed metabolic disorders (e.g., diabetes), or who experienced irregular menstrual cycles that prevented standardized scheduling were excluded.

Participants were instructed to avoid vigorous physical activity for 1 week before testing and to refrain from alcohol, caffeine, strong tea, and all medications for 48 h prior to each session. Diet records from the preceding week were collected, and participants were asked to maintain their habitual dietary patterns while avoiding high-fat and high-sugar foods during the experimental period to minimize dietary variability. They were required to maintain regular sleep schedules and avoid staying up late.

All tests were scheduled outside menstruation to avoid acute hormonal fluctuations associated with menstrual bleeding. To further reduce hormonal variability, exercise sessions were conducted approximately 1 week after the end of menstruation, corresponding to the early to mid-follicular phase. Written informed consent was obtained from all participants before participation.

### 2.2 Experimental control

For the fasting trial, participants completed an overnight fast of approximately 11–15 h, standardized by requiring them to finish their final meal between 7:00–8:00 p.m. the evening before testing; only water was permitted during the fasting period. For the post-breakfast condition, participants consumed a standardized breakfast (∼500 kcal: 60% carbohydrate, 20% protein, 20% fat) and commenced the exercise session 90 min later, corresponding to a stable postprandial phase characterized by elevated blood glucose and insulin levels.

All experimental sessions were conducted between 7:30 and 9:30 a.m. to minimize the influence of circadian variation on metabolism. Testing took place in a controlled laboratory environment with constant temperature (24 °C–26 °C) and humidity (40%–60%).

### 2.3 Experimental procedures

Each participant completed two exercise sessions separated by a 7-day washout period. On each test day, participants arrived at the laboratory between 7:30 and 9:30 a.m., rested in a seated position for 20 min, and performed a standardized warm-up. All exercise sessions were supervised by trained research staff who ensured correct execution and consistent intensity across participants. Before data collection, each participant received standardized instruction and performed a familiarization session in which all four Tabata movements were demonstrated and practiced to ensure proper technique, particularly for high-knees and burpees. They were then fitted with the cardiopulmonary monitoring equipment, and gas exchange data were recorded continuously throughout the session.

Gas exchange variables were measured using a portable metabolic analyzer (COSMED K5, COSMED Srl, Rome, Italy) operating in breath-by-breath mode. Before each test, the device was calibrated according to the manufacturer’s instructions, including flow calibration with a 3-L syringe and gas calibration using a certified reference mixture (16% O_2_, 5% CO_2_). Participants wore a well-fitted oronasal mask to ensure an airtight seal and minimize leakage during high-intensity movements. The breath-by-breath data were processed using the system’s built-in filtering procedures and averaged with a 10-s moving window to reduce variability caused by rapid ventilatory fluctuations during the interval protocol.

**FIGURE 1:**
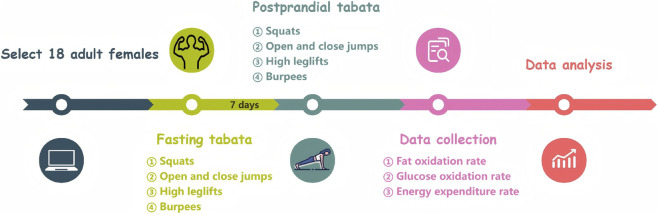
Experimental procedure chart (n = 18).

The Tabata protocol consisted of four exercises—squats, jumping jacks, high knees, and burpees. Each exercise was performed for 20 s followed by 10 s of rest, with the sequence repeated twice for a total duration of 4 min (Figure 1).

During the Tabata protocol, HR was recorded continuously using a Garmin chest-strap heart rate monitor (electrode-based sensor). The HR signal was transmitted wirelessly (ANT+/Bluetooth) to the COSMED K5 metabolic system and logged throughout the entire Tabata protocol. Data were exported from the COSMED system for analysis. Time-point HR values (30–240 s) represent elapsed time from the start of the Tabata protocol and were calculated as the mean HR over a 5-s window centered on each time point (t ± 2 s).

Rating of perceived exertion (RPE) was assessed using the Borg 6–20 RPE scale. Prior to testing, participants were familiarized with the scale and were instructed to report their overall perceived exertion (i.e., global effort including breathing difficulty and muscle fatigue), where six indicated “no exertion at all” and 20 indicated “maximal exertion.” RPE was obtained at each predefined time point (30–240 s; elapsed time from the start of the Tabata protocol). Specifically, participants provided a verbal RPE rating immediately after each 20-s exercise bout, during the subsequent 10-s rest period (i.e., at the end of each 30-s cycle), and the reported value was recorded by the investigator.

## 3 Measurements

### 3.1 Physiological indices measurement

BMI was determined as weight divided by height squared (kg/m^2^). The body fat percentage (BFP) was measured using a body composition tester. (InBody 720, Biospace Co., Ltd., Seoul, South Korea). VO_2_ (L/min) and VCO_2_(L/min) were continuously measured with the exercise cardiopulmonary tester (COSMED K5) during exercise. It is calculated that (Trombold et al., 2012; Frayn, 1983):

①Fat oxidation rate (g/min) = 1.695 × VO_2_ (L/min) - 1.701×VCO_2_ (L/min); ②Glucose Oxidation rate (g/min) = 4.585 × VCO_2_ (L/min) −3.2256 × VO_2_ (L/min); ③Energy consumption rate (kcal/min) = 3.716 × VO_2_ (L/min) +1.332 × VCO_2_ (L/min).

The fat oxidation rate, glucose oxidation rate, and energy consumption rate are all calculated using the 30-s value method. For each time period (e.g., 30s, 60s, 90s, etc.), the average values of oxygen consumption (VO_2_) and carbon dioxide production (VCO_2_) were used to estimate these rates. The data are not based on instantaneous peak values, but instead are derived from the average values over 30-s windows, providing a more stable and smooth estimation of metabolic rates.

### 3.2 Statistical analysis

Sample size estimation was performed using G*Power 3.1.9.7, assuming an effect size of 0.40, an alpha level of 0.05, and a statistical power of 0.95, indicating that a minimum of 10 participants was required. All statistical analyses were conducted using SPSS 26.0 (Chicago, IL, United States). Data are presented as mean ± standard deviation, and normality was confirmed using the Shapiro–Wilk test.

A two-way repeated-measures ANOVA (time × nutritional condition) served as the primary analytic model for fat oxidation, glucose oxidation, and energy expenditure. When the sphericity assumption was violated, the Greenhouse–Geisser correction was applied. Partial eta squared (η^2^_p_) was reported as the effect size, with thresholds of 0.04, 0.25, and 0.64 indicating small, medium, and large effects, respectively (Ferguson, 2009).

For variables showing a significant time × condition interaction (fat oxidation and glucose oxidation), *post hoc* simple effects analyses were conducted to compare the two nutritional conditions at each time point. p-values were adjusted using the Bonferroni correction to control for Type I error, and effect sizes for simple effects were expressed as Cohen’s d (Gülkesen et al., 2022).

For the variable without a significant interaction (energy expenditure), no simple effects analyses were performed; only descriptive statistics and the main effects from the ANOVA were reported.

To examine potential order or carryover effects inherent in the crossover design, exercise sequence (fasting-first vs. fed-first) was included as a between-subject factor. Condition × sequence interactions were tested for all metabolic outcomes, and no significant effects were observed, indicating that exercise order did not influence the results.

## 4 Results

### 4.1 Heart rate and Rating of Perceived Exertion (RPE)

Heart rate and Rating of Perceived Exertion (RPE) responses during the Tabata protocol are presented in Tables 1, 2. In both nutritional conditions, heart rate increased progressively from 30 s to 240 s, showing the typical cardiovascular response pattern of high-intensity interval exercise. No significant differences were observed between fasting and postprandial conditions at most time points (all p > 0.05), except for small differences at 30 s (p = 0.023), 150 s (p = 0.038), and 210 s (p = 0.031). However, these differences were minimal in magnitude (mean difference <3 bpm) and did not alter the overall heart rate trajectory.

**TABLE 1:** Heart rate responses during the Tabata protocol under fasting and postprandial conditions (beats/min).

Time (s)	Fasting HR (mean ± SD)	Postprandial HR (mean ± SD)	t-value	p-value
30s	110.32 ± 3.57	107.55 ± 4.72	2.51	0.023
60s	121.48 ± 3.28	119.54 ± 2.75	1.71	0.106
90s	134.85 ± 2.08	133.61 ± 3.23	0.49	0.629
120s	143.67 ± 2.32	141.24 ± 4.27	1.70	0.107
150s	150.92 ± 2.09	148.40 ± 2.69	2.25	0.038
180s	159.21 ± 3.91	157.09 ± 2.23	1.52	0.146
210s	168.43 ± 2.94	165.89 ± 2.87	2.35	0.031
240s	170.19 ± 3.53	168.20 ± 2.48	1.90	0.074

**TABLE 2:** RPE at each time point under fasting and postprandial condition.

Time (s)	Fasting RPE (mean ± SD)	Postprandial RPE (mean ± SD)	t-value	p-value
30s	11.03 ± 0.50	10.75 ± 0.53	2.03	0.059
60s	12.15 ± 0.41	11.95 ± 0.47	0.85	0.406
90s	13.48 ± 0.35	13.36 ± 0.42	0.14	0.89
120s	14.37 ± 0.37	14.12 ± 0.43	1.54	0.142
150s	15.09 ± 0.55	14.84 ± 0.58	0.15	0.88
180s	15.92 ± 0.45	15.71 ± 0.58	1.30	0.209
210s	16.84 ± 0.55	16.59 ± 0.34	1.03	0.317
240s	17.02 ± 0.49	16.82 ± 0.58	1.06	0.303

RPE also increased steadily over time in both trials. At all eight time points, fasting and postprandial RPE values did not differ significantly (all p > 0.05). The differences between conditions at each time point were small (generally <0.3–0.4 units), indicating comparable perceived exertion between trials.

Together, these findings indicate that both physiological (heart rate) and perceptual (RPE) exercise intensities were closely matched between fasting and postprandial conditions, ensuring that subsequent differences in substrate utilization reflect nutritional status rather than differences in exercise intensity (Tables 1, 2).

### 4.2 Time course of respiratory exchange ratio (RER)

**FIGURE 2:**
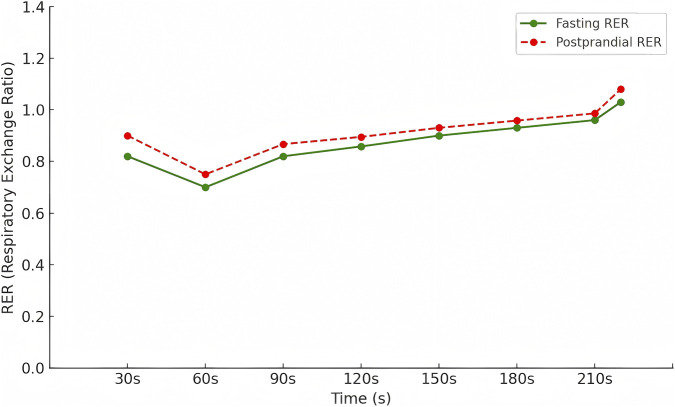
Respiratory exchange ratio (RER) responses during the Tabata protocol under fasting and post-breakfast conditions.

Figure 2 illustrates the time-course changes in RER during the Tabata exercise under fasting and postprandial conditions. In the early phase of the exercise (30–60 s), RER values remained below 1.0 in both conditions (30 s: 0.82 vs. 0.90; 60 s: 0.70 vs. 0.75), indicating that energy production was predominantly supported by aerobic metabolism. As the exercise progressed, RER increased steadily in both conditions (90 s: 0.82 vs. 0.867; 120 s: 0.858 vs. 0.895) and continued to approach 1.0 during the 150–210 s interval (150 s: 0.90 vs. 0.93; 180 s: 0.93 vs. 0.958; 210 s: 0.96 vs. 0.986), reflecting the increasing contribution of anaerobic metabolism during high-intensity intermittent exercise.

Notably, during the final high-intensity burst (approximately 220 s), RER exceeded 1.0 in both conditions (fasting ≈1.03; postprandial ≈1.08). This pattern suggests substantial involvement of anaerobic metabolism and additional CO_2_ production derived from bicarbonate buffering of exercise-induced acidosis. Under such non–steady-state metabolic conditions, VCO_2_ no longer solely reflects oxidative substrate metabolism, and the stoichiometric estimation of substrate oxidation based on VO_2_ and VCO_2_ becomes less reliable.

In light of these physiological characteristics, only the time intervals with RER ≤1.0 were included in subsequent substrate oxidation analyses to ensure a more physiologically valid interpretation of metabolic data.

### 4.3 Fat oxidation rate

A two-way repeated-measures ANOVA revealed significant main effects of time (F (6, 102) = 413.75, p < 0.001, η^2^_p_ = 0.96) and nutritional condition (F (1, 17) = 234.97, p < 0.001, η^2^_p_ = 0.93), as well as a significant time × condition interaction (F (6, 102) = 64.07, p < 0.001, η^2^_p_ = 0.79).

Given the significant interaction, *post hoc* simple effects analyses were performed to compare the two nutritional conditions at each time point. In the fasting condition, the fat oxidation rate increased from 0.32 ± 0.09 g/min at 30 s to a peak of 1.05 ± 0.18 g/min at 60 s, followed by a gradual decline to 0.12 ± 0.03 g/min at 210 s. In the postprandial condition, values were consistently lower, rising from 0.23 ± 0.07 g/min at 30 s to 0.61 ± 0.07 g/min at 60 s and decreasing to 0.04 ± 0.03 g/min at 210 s.

**FIGURE 3:**
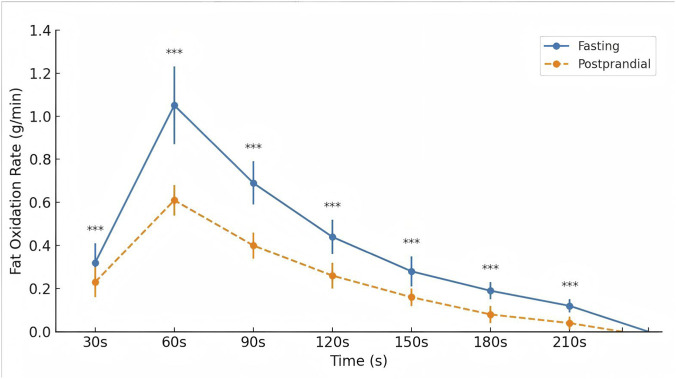
Fat oxidation rate of Tabata in fasting and postprandial states (g/min).*p < 0.05, **p < 0.01, ***p < 0.001.

Across all analyzed time points (30–210 s), simple effects analyses showed significantly higher fat oxidation in the fasting condition compared with the postprandial condition, with large effect sizes at each interval (Table 3; Figure 3).

**TABLE 3:** Simple effects analysis of fasting vs. postprandial fat oxidation (g/min).

Time (s)	Fasting fat (mean ± SD)	Postprandial (mean ± SD)	t-value	p-value	Mean difference (95% CI)	Effect size (Cohen’s d)
30s	0.32 ± 0.09	0.23 ± 0.07	6.08	<0.001	0.09 (0.06–0.13)	1.43
60s	1.05 ± 0.18	0.61 ± 0.07	14.34	<0.001	0.44 (0.37–0.50)	3.38
90s	0.69 ± 0.10	0.40 ± 0.06	11.46	<0.001	0.29 (0.24–0.34)	2.70
120s	0.44 ± 0.08	0.26 ± 0.06	9.07	<0.001	0.19 (0.14–0.23)	2.14
150s	0.28 ± 0.07	0.16 ± 0.04	7.26	<0.001	0.13 (0.09–0.16)	1.71
180s	0.19 ± 0.04	0.08 ± 0.04	10.12	<0.001	0.11 (0.09–0.14)	2.38
210s	0.12 ± 0.03	0.04 ± 0.03	14.52	<0.001	0.07 (0.06–0.09)	3.42

### 4.4 Glucose oxidation rate

A two-way repeated-measures ANOVA revealed significant main effects of time (F (7, 238) = 1015.24, p < 0.001, η^2^_p_ = 0.97) and nutritional condition (F (1, 21.33) = 136.42, p < 0.001, η^2^_p_ = 0.385), as well as a significant time × condition interaction (F (7, 28) = 4.61, p = 0.002, η^2^_p_ = 0.540).

Given the significant interaction, *post hoc* simple effects analyses were conducted to compare the two nutritional conditions at each time point. In the fasting condition, glucose oxidation increased steadily from 0.30 ± 0.05 g/min at 30 s to a peak of 3.38 ± 0.46 g/min at 150 s, followed by a slight decline to 3.14 ± 0.38 g/min at 240 s. In the postprandial condition, glucose oxidation was higher at all time points, rising from 0.48 ± 0.10 g/min at 30 s to a peak of 3.65 ± 0.52 g/min at 150 s, and decreasing to 3.18 ± 0.31 g/min at 240 s.

**FIGURE 4:**
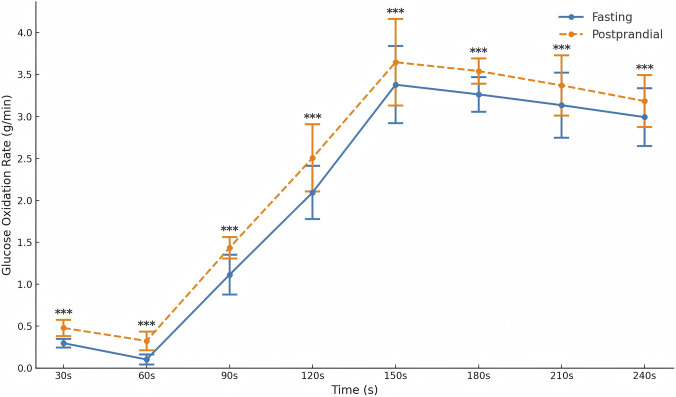
Glucose oxidation rate of Tabata in fasting and postprandial states (g/min).*p < 0.05, **p < 0.01, ***p < 0.001.

Across all measured time points, simple effects analyses confirmed that glucose oxidation was significantly higher in the postprandial condition than in the fasting condition (Table 4; Figure 4).

**TABLE 4:** Simple effects analysis of fasting vs. postprandial glucose oxidation (g/min).

Time (s)	Fasting fat (mean ± SD)	Postprandial (mean ± SD)	t-value	p-value	Mean difference (95% CI)	Effect size (Cohen’s d)
30s	0.30 ± 0.05	0.48 ± 0.10	9.22	<0.001	0.18 (0.22–0.14)	2.17
60s	0.10 ± 0.06	0.32 ± 0.11	9.41	<0.001	0.22 (0.27–0.17)	2.22
90s	1.11 ± 0.24	1.43 ± 0.13	8.39	<0.001	0.32 (0.40–0.24)	1.98
120s	2.09 ± 0.32	2.51 ± 0.40	5.90	<0.001	0.41 (0.56–0.27)	1.39
150s	3.38 ± 0.46	3.65 ± 0.52	5.50	<0.001	0.27 (0.37–0.17)	1.30
180s	3.26 ± 0.21	3.54 ± 0.15	10.29	<0.001	0.28 (0.33–0.22)	2.42
210s	3.13 ± 0.39	3.37 ± 0.36	10.98	<0.001	0.24 (0.28–0.19)	2.59
240s	2.99 ± 0.35	3.18 ± 0.31	7.96	<0.001	0.19 (0.24–0.14)	1.88

### 4.5 Energy expenditure rate

A two-way repeated-measures ANOVA revealed a significant main effect of time on energy expenditure (F (2.89, 98.13) = 1019.52, p < 0.001, η^2^_p_ = 0.97) and a significant main effect of nutritional condition (F (1, 34) = 24.35, p < 0.001, η^2^_p_ = 0.42). The time × condition interaction was not significant (F (7, 28) = 1.02, p = 0.443, η^2^_p_ = 0.20), indicating that both conditions followed a similar temporal pattern.

In the fasting condition, energy expenditure increased from 2.37 ± 0.22 kcal/min at 30 s to a peak of 9.70 ± 0.39 kcal/min at 120 s, and then declined to 6.87 ± 0.35 kcal/min at 240 s. In the postprandial condition, values were higher overall, rising from 3.13 ± 0.31 kcal/min at 30 s to 10.18 ± 0.29 kcal/min at 120 s and decreasing to 7.15 ± 0.42 kcal/min at 240 s.

**FIGURE 5:**
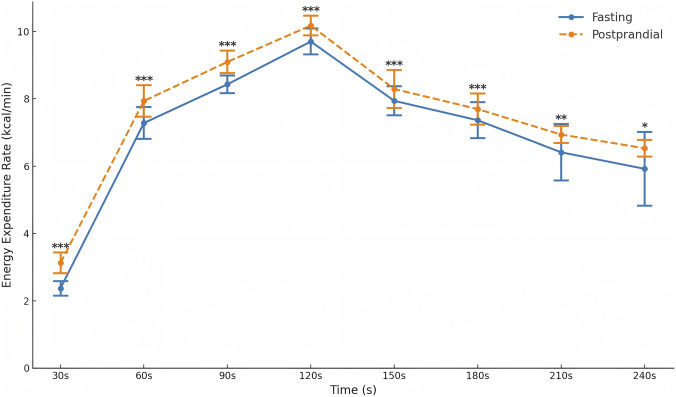
Energy expenditure rate of Tabata in fasting and postprandial states (g/min).*p < 0.05, **p < 0.01, ***p < 0.001.

Across all time points, energy expenditure was significantly higher in the postprandial condition compared with the fasting condition, while both conditions exhibited the same time-dependent trajectory (Table 5; Figure 5).

**TABLE 5:** Descriptive statistics of energy expenditure during Tabata under fasting and postprandial conditions (kcal/min).

Time (s)	Fasting (mean ± SD)	Postprandial (mean ± SD)
30s	2.37 ± 0.22	3.13 ± 0.31
60s	7.28 ± 0.47	7.94 ± 0.47
90s	8.43 ± 0.27	9.10 ± 0.33
120s	9.70 ± 0.39	10.18 ± 0.29
150s	7.94 ± 0.43	8.29 ± 0.56
180s	7.36 ± 0.54	7.69 ± 0.47
210s	6.41 ± 0.84	6.94 ± 0.26
240s	5.92 ± 1.09	6.53 ± 0.25

## 5 Discussion

### 5.1 Acute effects of fasting on fat oxidation

This study showed that fat oxidation was markedly higher in the fasting condition during the early stages of the Tabata protocol, with the greatest difference observed at 60 s (1.05 ± 0.18 g/min vs. 0.61 ± 0.07 g/min). Such elevations are consistent with the metabolic profile of fasting—lower blood glucose and insulin concentrations, greater sensitivity to catecholamines, and increased activation of hormone-sensitive lipase—which together enhance lipolysis and increase the supply of free fatty acids for oxidation (Frühbeck et al., 2014; Wang et al., 2008; Proença et al., 2024).

Reduced hepatic glycogen is another likely contributor. Low liver glycogen availability in the morning fasting state has been shown to shift substrate preference toward fat oxidation through AMPK activation and greater reliance on mitochondrial β-oxidation. The present findings support this idea by showing that the initial metabolic response during a high-intensity protocol is strongly shaped by substrate availability at the onset of exercise.

Although high-intensity exercise is typically associated with increased carbohydrate dependence, several studies have reported elevated fat oxidation during fasting even at moderate to high intensities (Achten and Jeukendrup, 2004; Pélissier et al., 2024; Chávez-Guevara et al., 2023). Our results extend these observations by showing that very short intermittent bouts, such as those used in Tabata training, may permit transient periods where fat oxidation remains relatively elevated before the expected shift toward carbohydrate utilization occurs.

As the intervals progressed, fat oxidation declined steadily, consistent with the increasing anaerobic contribution and greater glycolytic demand characteristic of high-intensity efforts (Atakan et al., 2022; González-Gálvez et al., 2024). Taken together, these results emphasize the acute influence of fasting on substrate selection during a single 4-min session. These findings should not be interpreted as evidence of long-term changes in body composition or fat loss.

### 5.2 Glucose oxidation and energy expenditure responses in the postprandial state

In contrast to the fasting trial, glucose oxidation was significantly higher at all time points in the postprandial condition, peaking at 150 s (3.65 ± 0.52 g/min). Total energy expenditure was also consistently higher. These responses reflect the postprandial metabolic environment, where elevated glucose and insulin levels enhance carbohydrate availability and promote glucose uptake through increased GLUT4 translocation (Kang et al., 2023; Borror et al., 2018).

This pattern aligns with previous work showing that beginning high-intensity exercise in a fed state leads to greater glycolytic flux, faster glycogen breakdown, and stronger sympathetic activation (Bergouignan et al., 2006; Winding et al., 2019). Higher muscle glycogen stores after feeding may also help maintain performance across repeated intervals and delay fatigue.

The elevated energy expenditure in the postprandial condition may reflect both higher metabolic turnover and enhanced adrenergic stimulation. Although these findings may have implications for energy balance or glycemic regulation, such interpretations remain speculative. The present study evaluated only acute metabolic responses and did not assess extended post-exercise energy expenditure or 24-h energy balance.

It is also worth noting that the structure of Tabata training—brief, repeated bouts of intense effort—demands rapid access to readily available fuels. This may amplify differences in substrate selection between feeding conditions, helping explain why carbohydrate use remained consistently higher in the postprandial trial (Sylow et al., 2017; Brun et al., 2022).

### 5.3 Mechanistic interpretation and metabolic flexibility between conditions

The distinct substrate utilization patterns observed between the fasting and postprandial conditions can be explained by well-established metabolic regulatory mechanisms. In the fasting state, reduced insulin and elevated glucagon levels promote adipose tissue lipolysis, increasing the availability of circulating free fatty acids for oxidation. Concurrent activation of AMPK further facilitates fatty acid transport into mitochondria and enhances β-oxidation (Watt et al., 2009; Lundsgaard et al., 2018; Kido et al., 2023). Lower hepatic and potentially lower muscle glycogen stores may also shift the metabolic preference toward fat utilization during the initial stages of exercise (Muscella et al., 2020).

In contrast, the postprandial condition is characterized by elevated insulin concentrations that suppress lipolysis while markedly enhancing glucose uptake and utilization through GLUT4-mediated pathways (Gannon et al., 2015; Richter and Hargreaves, 2013). Higher carbohydrate availability, combined with increased sympathetic activation after feeding, promotes glycogen breakdown and glycolytic flux, particularly during high-intensity efforts (Zsombok et al., 2024; Eglseer et al., 2023). As a result, carbohydrate oxidation becomes the predominant pathway throughout the Tabata protocol in the fed state. These contrasting responses highlight the concept of metabolic flexibility, referring to the body’s ability to adjust fuel selection based on current hormonal and nutritional conditions. This capacity enables the body to efficiently meet the energetic demands of high-intensity exercise in both fasting and fed states.

The present findings reflect acute metabolic responses from a single 4-min Tabata session and do not provide evidence for long-term changes in body composition, fat loss, or metabolic adaptation. Whether these short-term substrate-use patterns have cumulative effects remains unknown. Another consideration is that participants were untrained, and differences in movement efficiency—especially during high-knees and burpees—may have influenced metabolic outcomes. Future work should include extended post-exercise measurements, comparisons of different HIIT formats and training volumes, and evaluations in both trained and untrained populations, as well as longer-term interventions to determine how repeated Tabata training under different nutritional states shapes metabolic and physiological adaptations.

## 6 Conclusion

This study demonstrated that fasting and post-breakfast conditions elicit distinct acute metabolic responses during a short, high-intensity Tabata protocol in women. Fat oxidation was consistently higher in the fasting state, whereas glucose oxidation and total energy expenditure were higher in the post-breakfast state throughout the exercise. These findings highlight that pre-exercise nutritional status plays a significant role in shaping substrate utilization and energy expenditure during high-intensity interval exercise, reflecting the body’s capacity to rapidly adjust fuel selection according to immediate metabolic and hormonal conditions.

## Data Availability

The raw data supporting the conclusions of this article will be made available by the authors, without undue reservation.
